# Actinomycosis masquerading as a nasal polyp – a rare entity and diagnostic challenge

**DOI:** 10.3205/dgkh000520

**Published:** 2024-12-16

**Authors:** Revathy Sakthimohan, Ajitha Rajalingam, Thanka Johnson, K. Vivek Rajan

**Affiliations:** 1 Department of Pathology, Sree Balaji Medical College & Hospital, Chromepet, Chennai, Tamil Nadu, India; 2 Department of ENT, Sree Balaji Medical college Chromepet, Chennai, Tamil Nadu, India

**Keywords:** Actinomycosis, Actinomyces israelii, nasal polyp, granulomatous disease, case report

## Abstract

Actinomycosis is an endogenous bacterial infection caused by *Actinomyces israelii*. This bacterium reside on the mucosa of oral cavity, tonsils, and genitourinary tract. Any insult such as trauma, surgery, or foreign body disrupts the mucosal barrier and gives entry to the underlying tissue to cause disease.

We describe a rare presentation of Actinomycosis presenting as a nasal polypoidal mass in a young female, an uncommon presentation, thereby causing diagnostic challenge as it may mimic other lesions with similar clinical presentations like fungal polyps, allergic polyps or chronic sinusitis. A 22 year old female presented with history of recurrent upper respiratory tract infection to the ENT Outpatient Department, clinical evaluation showed deviated nasal septum and radiology showed a nasal polyp and patient underwent submucosal resection with middle meatal antrostomy and micro-debrider assisted polypectomy. Histopathology showed respiratory epithelium with underlying stroma showing dense basophilic filamentous organisms surrounded by peripheral eosinophilic clubs that were Gram-positive and Gomori Methanamine Silver stain positive. The disease was diagnosed as Actinomycosis. The disease is a mimicker of various diseases such as nocardiosis, botryomycosis or tuberculosis having a wide range of symptoms and affecting multiple organs. Prevalence of actinomycosis in nasal region is rare thereby increasing the risk of misdiagnosis. Thus, Actinomycosis should be kept as a differential diagnosis in any chronic infectious diseases of the nasal cavity and monitored closely to ensure precise diagnosis and timely management.

## Abstract (de)

Aktinomykose ist eine endogene bakterielle Infektion, die durch *Actinomyces israelii* verursacht wird. Der Erreger siedelt auf der Schleimhaut der Mundhöhle, der Tonsillen und des Urogenitaltrakts. Jede Verletzung, z. B. Trauma, ein chirurgischer Eingriff oder ein Fremdkörper, stört die Schleimhautbarriere, ermöglicht das Eindringen in das darunter liegende Gewebe und kann eine Erkrankung verursachen.

Beschrieben wir eine seltene Actinomykose, die sich bei einer jungen Frau als polypöse Masse in der Nase darstellt. Das ist eine ungewöhnliche Situation, die eine diagnostische Herausforderung darstellt, da sie andere Läsionen mit ähnlichen klinischen Symptomen wie Pilzpolypen, allergische Polypen oder chronische Sinusitis imitieren kann. Eine 22-jährige Frau stellte sich mit einer Vorgeschichte rezidivierender Infektionen der oberen Atemwege in der HNO-Ambulanz vor. Die klinische Untersuchung ergab eine Nasenscheidewandverkrümmung und die Radiologie einen Nasenpolypen, woraufhin sich die Patientin einer submukösen Resektion mit mittlerer Meatusantrostomie und einer mikrodebrierten Polypektomie unterzog. Die Histopathologie ergab ein respiratorisches Epithel mit darunter liegendem Stroma, das dichte basophile, fadenförmige Organismen aufwies, die von peripheren eosinophilen Keulen umgeben waren, die Gram-positiv und Gomori-Methanamin-Silberfärbung positiv waren. Die Krankheit wurde als Aktinomykose diagnostiziert. Diese Krankheit ahmt verschiedene Krankheiten wie Nocardiose, Botryomykose oder Tuberkulose nach, hat ein breites Spektrum an Symptomen und befällt mehrere Organe. Die Prävalenz der Actinomykose im Nasenbereich ist selten, was das Risiko einer Fehldiagnose erhöht.

## Introduction

Actinomycosis is a chronic, endogenous, granulomatous infectious disease [1] caused by *Actinomyces israelii*. The bacterium was first described in 1896 and was originally named as *Streptothrix israelii*. Actinomyces is a Gram-positive anaerobic bacillus which is currently a family of 26 different species which are obtained from the normal human microbiota [2]. These organisms are commensals of the oral mucosa, tonsils, and also in the female genitourinary tract [3]. Any trauma, surgery, or foreign body disrupts the mucosal barrier and gives entry to the underlying tissue to cause disease [4]. Thoracic actinomycosis is associated with conditions such as alcohol use disorder. Pelvic actinomycosis is linked to use of intrauterine devices [5]. This infection is common in areas with low socioeconomic status. The disease is known for complications such as spread of infection, brain abscess, bony involvement, meningitis and occasionally also disseminated actinomycosis. 

Actinomycosis is either overlooked, or lately diagnosed and leads to delay in treatment causing significant morbidity and mortality. Nasal polypoidal presentation of actinomycosis is an extremely uncommon finding. We describe a rare case of actinomycosis presenting as a polypoidal nasal mass in a young female and confirmed on histopathology, it is a uncommon disease, causing diagnostic challenge. Histopathology plays a pivotal role in such cases so as to aid in faster patient management with systemic antibiotic therapy.

## Case report

**Figure 1:**
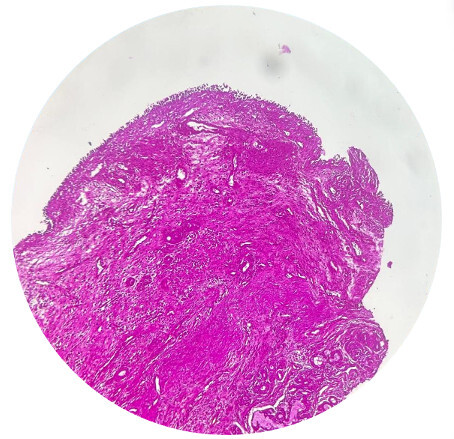
Respiratory epithelium with dense chronic inflammation in underlying stroma (H&E;10x)

**Figure 2:**
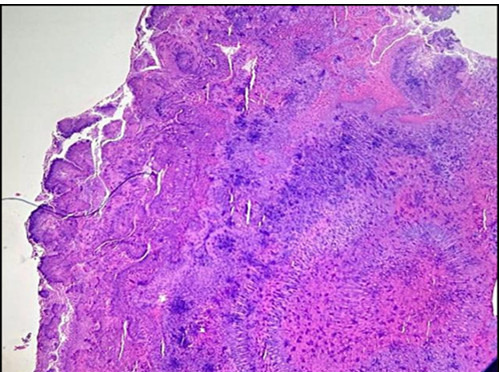
Actinomycotic sulphur granules (H&E;40x)

**Figure 3:**
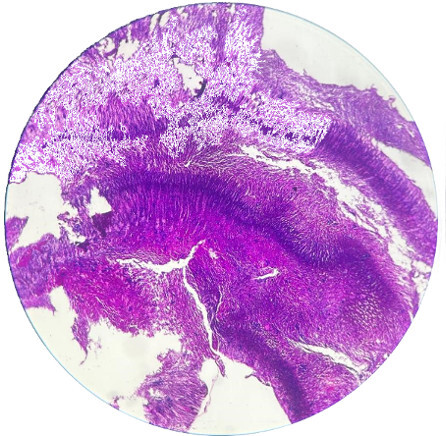
Basophilic filamentous organisms representing Actinomyces (Gramsstain; 40x)

**Figure 4:**
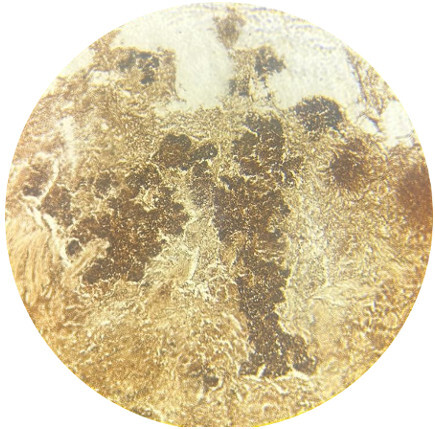
Black colour stained actinomyces organisms on Gomori methanamine silver stain (10x)

A 22-year-old female presented to the ENT outpatient department with right sided headache, nose block and recurrent upper respiratory tract infection for two months that was insidious in onset and intermediate in nature. Patient also complained of heaviness of head and frequent nasal discharge. she also gave a history of hemoptysis two weeks ago. Her general physical examination was normal. She had no other significant comorbidities. Local examination showed pink nasal mucosa and deviated nasal septum to left. Radiological examination (CT paranasal sinuses) showed polypoidal mucosal thickening in right maxillary and right anterior ethmoidal sinus, widening of right osteomeatal complex. Mild mucosal thickening of left frontal and bilateral ethmoid sinuses, suggestive of sinusitis was noted. Right inferior turbinate hypertrophy was also found. She was clinically diagnosed with deviated nasal septum to left and chronic sinusitis. After preoperative evaluation, patient underwent submucosal resection with micro-debrider assisted polypectomy and the representative tissue bits from right maxillary and ethmoidal sinus were sent for histopathological examination. Grossly, multiple grey white soft tissue bits altogether amounting to 1cc was received in histopathology. Microscopic examination showed fragments of tissues lined by respiratory pseudostratified ciliated columnar epithelium with dense chronic inflammatory cells in the underlying stroma (Figure 1 (Fig. 1)). Fungal organisms showing actinomycotic sulphur granules (Figure 2 (Fig. 2)). Dense basophilic filamentous organisms surrounded by eosinophilic clubs, known as Splendor hoeppli phenomenon, were noted within dense mixed inflammatory cells. Gram stain and Gomori methanamine silver stains were done. Gram stain showed Gram-positive intertwined branching filamentous organisms with radially arranged peripheral hyphae (Figure 3 (Fig. 3)). Gomori methanamine silver stain showed colony of filamentous micro-organisms which stained black (Figure 4 (Fig. 4)). Histopathology and special stains confirmed the diagnosis of actinomycosis. Patient was given intravenous antibiotics, cephalosporin for a week and given oral antibiotics for 3 months with periodic follow up. Patient is currently doing well with no evidence of recurrence or infection.

## Discussion

Actinomycosis is a chronic granulomatous disease, causing wide range of human infections. These infections present as hard mass causing a clinical dilemma in diagnosis. Actinomyces is part of the commensals in the oral cavity and vaginal flora of healthy individuals and become pathological only in immunocompromised conditions or invasion into injured tissue [6]. Histopathology combined with microbiological examination provides accuracy in the diagnosis of these lesions. However, usage of antimicrobials before sampling for culture, inhibition of growth by other contaminant organisms may give rise to false negative results, promoting the importance of histopathology. Molecular detection methods and identification techniques are widely used for the diagnosis in recent era. The most sensitive method of identification is rRNA gene sequencing [7]. 

The infection can affect any part of the body, while common anatomical regions include cervicofacial (50–60%), pulmonary (15%), abdominopelvic (25%), and cerebral areas. Cervicofacial disease accounts for 55% of all infections, majority as intraoral and periapical forms. Disseminated infections may occur in chest, abdomen, lungs, liver, central nervous system and uterus [8]. Actinomycosis presents with pain, swelling, change in colour, induration, abscess, fistulas. Actinomycosis of nasal cavity resembles sinusitis presenting with nasal obstruction, purulent discharge, sensation of pressure and facial swelling [9].

Microscopy shows chronic granulomatous infection along with masses of branching Gram-positive filamentous aggregates of actinomyces organisms. Small yellow clumps, ranging from 0.1 to 1 mm in diameter, referred to as sulfur granules are seen. This mass is surrounded by neutrophils and peripheral eosinophilic clubs that is stabilized by a protein-polysaccharide complex which provides resistance to host defence mechanism by inhibiting phagocytosis [10] .This is formed because of the antigen-antibody response, which can also be observed in fungal infections (such as zygomycosis, sporotrichosis etc.), bacterial infections (such as botryomycosis, nocardiosis etc.), parasitic infestations (such as strongyloides, schistosomiasis etc.). Non-infectious conditions such as atopy and hypereosinophilic syndrome can also cause this phenomenon. Gram stain is a sensitive method of detection which shows Gram-positive filamentous branching bacteria at the periphery. This stain has higher sensitivity even in cases with prior antibiotic therapy. On Gram stain these organisms occur as non-spore forming Gram-positive rods. Another species named *Actinomyces meyeri* appears as small and non-branching forms, while all other forms appear as branching filamentous rods [11]. 

Culture of actinomyces is a slow process; it may need up to 15–20 days. Hence, incubation of at least 10 days is necessary before reporting of a negative culture. However, routine histopathology and special stains like Gram staining and Gomori’s methanamine silver (GMS) staining on biopsy can provide an exact diagnosis at a much lesser duration of time. 

Actinomycosis of nasal cavity is extremely rare and should be considered a differential for chronic sinusitis due to other nasal infections. In a study by Park et al. [12] on actinomycosis of the nasal cavity, 11 cases were included out of which the common symptom was purulent nasal discharge. Endoscopic surgery along with oral and intravenous antibiotics like penicillin for weeks to months treats the condition. Tetracyclines or erythromycin is administered to patients who are allergic to penicillin. The prognosis of nasal actinomycosis remains good with symptoms improving with antibioticsp [13]. 

In our current case, young female patient presented with symptoms of nasal polyp which clinically mimicked fungal polyp or inflammatory nasal polyp. Histopathology and Gram staining confirmed the diagnosis of actinomycosis of nasal cavity. Culture could not be done due to the anaerobic nature of the organism. Bacterial culture was not done as culture remains sterile in about 50% of cases. Patient was started on intravenous antibiotics for a week followed by oral antibiotics for three months and is currently under follow up.

## Conclusion

Actinomycosis is a chronic, rare, granulomatous disease caused by a diverse species of actinomyces organisms. It can manifest in atypical forms, such as presenting as a nasal polyp, which highlights the importance of considering rare infectious etiologies in the differential diagnosis of nasal masses. Actinomycosis is a mimicker of various diseases having wide range of symptoms and affecting multiple organs. In our case, actinomycosis presented as nasal polyp which is extremely uncommon and should be kept as a differential diagnosis in any chronic infectious nasal diseases and carefully observed for prompt diagnosis and improved patient outcome by timely antibiotic therapy.

## Notes

### Competing interests

The authors declare that they have no competing interests.
